# The use of technology in the subcategorisation of osteoarthritis: a Delphi study approach

**DOI:** 10.1016/j.ocarto.2020.100081

**Published:** 2020-06-09

**Authors:** Claire Mennan, Timothy Hopkins, Alastair Channon, Mark Elliott, Brian Johnstone, Timor Kadir, John Loughlin, Mandy Peffers, Andrew Pitsillides, Nidhi Sofat, Caroline Stewart, Fiona E. Watt, Eleftheria Zeggini, Cathy Holt, Sally Roberts, The OATech Network+ Consortium

**Affiliations:** a The Robert Jones & Agnes Hunt Orthopaedic Hospital NHS Foundation Trust & School of Pharmacy & Bioengineering, Keele University, Oswestry, Shropshire, SY10 7AG, UK; b School of Computing & Mathematics, Keele University, Staffordshire, ST5 5BG, UK; c Institute of Digital Healthcare, WMG, University of Warwick, Coventry, CV4 7AL, UK; d Department of Orthopaedics and Rehabilitation, Oregon Health and Science University, Portland, OR, 97239, USA; e Optellum Ltd, Oxford Centre for Innovation, Oxford, OX1 1BY, UK; f Biosciences Institute, International Centre for Life, Newcastle University, Newcastle upon Tyne, NE1 3BX, UK; g Institute of Ageing and Chronic Disease, The University of Liverpool, L69 7ZX, UK; h Comparative Biomedical Sciences, The Royal Veterinary College, London, NW1 0TU, UK; i Institute of Infection and Immunity, St Georges University of London, SW17 0RE, UK; j Centre for Osteoarthritis Pathogenesis Versus Arthritis, Kennedy Institute of Rheumatology, NDORMS, University of Oxford, OX3 7FY, UK; k Helmholtz Zentrum München - German Research Center for Environmental Health, Institute for Translational Genomics, Ingolstädter Landstr., 185764, Neuherberg, Germany; l Cardiff University, Queen's Buildings, The Parade, Cardiff, CF24 3AA, UK

**Keywords:** Stratification, Osteoarthritis, Technology, Phenotype, Omics, Biomarkers

## Abstract

**Objective:**

This UK-wide OATech Network + consensus study utilised a Delphi approach to discern levels of awareness across an expert panel regarding the role of existing and novel technologies in osteoarthritis research. To direct future cross-disciplinary research it aimed to identify which could be adopted to subcategorise patients with osteoarthritis (OA).

**Design:**

An online questionnaire was formulated based on technologies which might aid OA research and subcategorisation. During a two-day face-to-face meeting concordance of expert opinion was established with surveys (23 questions) before, during and at the end of the meeting (Rounds 1, 2 and 3, respectively). Experts spoke on current evidence for imaging, genomics, epigenomics, proteomics, metabolomics, biomarkers, activity monitoring, clinical engineering and machine learning relating to subcategorisation. For each round of voting, ≥80% votes led to consensus and ≤20% to exclusion of a statement.

**Results:**

Panel members were unanimous that a combination of novel technological advances have potential to improve OA diagnostics and treatment through subcategorisation, agreeing in Rounds 1 and 2 that epigenetics, genetics, MRI, proteomics, wet biomarkers and machine learning could aid subcategorisation. Expert presentations changed participants’ opinions on the value of metabolomics, activity monitoring and clinical engineering, all reaching consensus in Round 2. X-rays lost consensus between Rounds 1 and 2; clinical X-rays reached consensus in Round 3.

**Conclusion:**

Consensus identified that 9 of the 11 technologies should be targeted towards OA subcategorisation to address existing OA research technology and knowledge gaps. These novel, rapidly evolving technologies are recommended as a focus for emergent, cross-disciplinary osteoarthritis research programmes.

## 1 Introduction

It is predicted that there will be a 4- to 6-fold increase in the number of total joint replacements for osteoarthritis (OA) in the coming decades [1]. Despite the increase in prevalence and the large body of literature existing on the subject, definitions of OA subcategories, whether in clinical or research environments, are often disparate. The OATech Network+, a multidisciplinary consortium, had identified this as a potential limitation to furthering OA research. Whilst X-rays have been one of the most commonly used technologies for studying OA for decades, there have been many recent technological developments applied to the field, for example, in genomics and other ‘omics’, different forms of imaging, and computational analysis of big data.

The OATech Network + organised a consensus meeting combining experts in a broad range of existing and novel technologies (with basic scientists and clinicians) to appraise the potential of existing and new technologies and improve OA subcategorisation. A Delphi approach was adopted, aiming to recommend improved targeting of technology for OA subcategorisation so that existing and emerging treatments could be applied more effectively to selected patients or subgroups.

The meeting commenced with experts in the fields of engineering, rheumatology, orthopaedic surgery, radiology, physiotherapy, biology and OA pain perception sharing their experience of OA research. Experts in more recently developed technologies lectured on their OA research application, summarised below.

### 1.1 Genetics and genomics

The field of complex trait genetics has witnessed a revolution in technological advances over the last decade, enabling the genome-wide interrogation of sequence variation, leading to the discovery of thousands of genetic risk loci. Recent methodological advances have also enabled deep molecular characterisation of disease-relevant tissues collected from human patients or studied in cellular and organismal models of disease. Together, these can help enhance our understanding of the mechanisms underlying disease development and progression [2]. Large-scale genetics can help improve our understanding of the genetic aetiology of OA and related sub-groups by interrogating big data in genetics, genomics and medically-relevant phenotypes from rich epidemiological resources, patient collections and disease registries. Functional genomic approaches for integrated molecular phenotyping of relevant cell types can help translate insights from genomics into mechanisms of disease in order to overcome the critical barrier of there being currently no disease-modifying treatment for OA. The relevant diseased OA tissues are readily available from joint replacement surgery, enabling the study of molecular processes in the appropriate tissues, both to fill a gap in our fundamental understanding of biology and to identify novel therapeutic avenues.

### 1.2 Epigenetics and functional analysis

Epigenetics is a mechanism used by the cell, tissue and organ to regulate gene expression in a dynamic manner by reversible chemical changes to the genome. There are three epigenetic markers: DNA methylation, histone modification and the activity of regulatory RNAs [3]. Epigenetic changes are context specific and show temporal and spatial effects. They act during skeletogenesis and joint formation, and have a role in OA [[3], [4], [5]]. As for genomic studies, the diseased joint tissues such as articular cartilage, synovium or bone, are used in relatively large quantities to extract DNA, chromatin and RNA for epigenetic analysis. Such studies have led to subcategorisation of OA by, for example, identifying individuals who appear to have an inflammatory component to their disease [4].

### 1.3 Proteomics and metabolomics

Proteomics and metabolomics can be used to identify molecules as possible predictors of early disease, disease progression and response to treatment. Synovial fluid contains systemic proteins and metabolite markers of disease and holds significant potential for the discovery of proteins and metabolites to aid subcategorisation of the disease.

Whilst transcriptomics can indicate the proteome, the relationship between mRNA and proteins is complex and thus identifying proteins in a sample and how they vary is paramount. Quantitative proteomic differences between sample groups can be identified using either absolute or relative quantification, with or without labelling (reviewed [6]). Absolute quantification has been used to measure up to 20 targeted proteins in a single experiment [7]. Label-free relative quantification using synovial fluid has been used and predictors of treatment outcome with autologous chondrocyte implantation (ACI) have been investigated for a number of biomarkers [8]. Nuclear magnetic resonance (NMR) and MS have been used in assessing metabolomics, being non-destructive, quantitative, reproducible and cost effective. Both techniques have identified up to 32 differentially expressed metabolites in synovial fluid from OA and rheumatoid arthritis [9]^.^

Degradomics is another proteomic method that may be useful in OA subcategorisation, assessing cleavage products at different stages in OA [8]. A further development, Matrix Assisted Laser Desorption Ionization Mass Spectrometry Imaging (MALDI-IMS), has been used to identify proteins and neopeptides altered in cartilage ageing and OA [8].

### 1.4 Molecular signatures and biomarkers

All the above techniques (genomics, epigenomics, proteomics) can assist in the search for OA biomarkers, in terms of the “Burden of disease, Investigative, Prognostic, Efficacy of intervention and Diagnostic (BIPED)” classification scheme [10]. To date, many candidate proteins, carbohydrates and lipids [11] have been investigated [12]. Several are associated with disease progression in OA cohorts, but are not able to stratify individuals [13]. A ‘molecular signature’ representing multiple protein or non-protein markers may be more realistic for OA than finding a single biomarker, perhaps better indicating relevant shared mechanisms within the disease.

Although singleplex antibody-based assays remain the mainstay for investigation of candidate protein biomarkers, multiplexing with higher sensitivity and specificity for complex biological fluids is now possible by proprietary adaptive immunoassay approaches, such as electrochemiluminescence or proximity extension assays (combining antibody and PCR technology) [14]. Whether using immunoassay or mass cytometry (e.g. CyTOF), antibodies limit the absolute number and combinations possible, whereas non-antibody approaches circumvent these issues. Modified aptameric assays (aptamers being short sequences of nucleotides which are selected for their specificity to bind proteins in much the same way as an antibody) can be multiplexed to quantify thousands of proteins simultaneously in a single sample. These approaches have the ability to identify molecular endotypes (molecular subgroups in disease) or to predict drug toxicity and transform the way we are able to dissect molecular pathways or identify molecular signatures as biomarkers in biological fluids.

### 1.5 Clinical engineering

The International Classification of Functioning, Disability and Health (ICF) provides a framework for understanding disability which links the body functions and structures to activity and participation. Clinical movement analysis, in particular 3D gait analysis, allows clinicians to measure the impact of OA on walking. This is important as patients often perceive their walking pattern as a cause as well as a consequence of the disease. Patients with unilateral disease often develop bilateral symptoms [15].

Previous work [16] has described gait in patients with single joint disease, who do not have a typically antalgic gait pattern, but have knee loading which is high throughout the stance phase, giving them a high moment impulse, combined with muscular co-contraction. This co-contraction, measured using electromyography (EMG) further increases contact forces in the joint. 3D gait analysis can detect bilateral overloading in both hip and knee joints in patients with unilateral, single joint disease. The adopted tentative gait pattern seems to predispose other joints to OA.

Whilst knee pain and loading measures improve after knee arthroplasty, some patients improve more than others and abnormal loading patterns often persist [16]. 3D gait analysis is useful in understanding the control and loading of the joints during movement and interpreting how these change in OA gait is important in providing appropriate therapies, such as bracing or biofeedback.

In knee OA populations biomechanical measures at baseline have also been used to predict radiographic disease progression [17], future total knee arthroplasty (TKA) [18] and stratify response to interventions such as lateral wedge insoles and TKA [16].

### 1.6 Activity monitoring

Recent OARSI guidelines have advocated the use of activity monitoring devices to collect objective measures of physical activity [19]. It is important for individuals with OA to remain physically active. Evidence indicates that it can reduce OA related pain, in addition to increasing muscle strength, joint range of motion and cardiovascular fitness [20]. Physical activity levels measured in OA populations over the longer term (3–12 months post-surgery) show no substantial increases in activity after 12 months [21]. Therefore more behavioural interventions are required to promote physical activity in the recovery period, a conclusion that could be missed when using more subjective self-reported measures.

Activity monitoring technology is rapidly advancing but for subgrouping of OA requires large amounts of data. Smart phones and wearable technology now offer the potential to collect this data outside of the laboratory and unobtrusively.

### 1.7 Machine learning and ‘big data’

Much of the technology described with potential to improve OA stratification creates very large data sets which require computational analysis; as the quantity of data increases, meaningful analysis becomes more challenging. The use of complex artificial neural network architectures or machine learning (ML) have been shown to be capable of representing and learning predictable relationships in many diverse types of data. These computational tools hold promise for transforming the future of ‘omics’ and other technologies which acquire huge data sets or 'big data' [22].

Imaging modalities such as MRI are used as clinical diagnostic tools and contain vast amounts of information which lend themselves well to analysis via ML. In the following example, ML is applied to image analysis of OA in the spine, thus demonstrating the potential value of this technology in identifying subgroups of OA. ML has been used to develop an automated method for grading degeneration in the spine and intervertebral disc on MRIs [23,24], as used in the Pfirrmann Score [25] for degenerative disc disease or OA of the spine (developed as ‘SpineNet’). The system can robustly extract measurements for this, in addition to having the potential to identify other phenotypes such as spinal stenosis or ‘Modic’ changes in the vertebral endplates. This approach requires well defined cohorts of patients with appropriate levels of consent for this type of data storage and analysis, both for developing the program and then subsequently independent cohort(s) for validation. SpineNet also has the capability of producing so-called ‘Hotspots’ or saliency images that can be used to visualize the parts of the MRI that are the likely source of the output [23], so possibly defining completely new phenotypes from this unbiased approach.

A prerequisite for imaging biomarker discovery is the extraction of robust and discriminative radiological measurements from joint MRIs; however, the lack of imaging biomarker standardisation within the research community, the inherent intra- and inter-reader variability and time and cost has hampered research to date. Clearly ML is providing a powerful tool to aid in the analysis of ‘big data’ and medical images with diverse applications too numerous to discuss here. Future ML, computational analysis and the development of automated programs, can offer robust, repeatable and rapid analysis of large datasets (MRI images or any other potential ‘biomarker’) and provide important tools for subcategorisation and identification of OA biomarkers. As novel markers of OA emerge across the biological, biomechanical, clinical and imaging interfaces, their combination will provide increasingly powerful datasets and opportunities for ML applications across OA diagnostics and classification domains.

In summary, the technologies mentioned above have developed rapidly in the last decade. For example, a literature search for ‘genomics’ or ‘epigenomics’ (using Medline and Embase) over the last 30 years highlights the increased awareness and use of such technology. From 1990 to 1999 genomics or epigenomics shows a total of 10 publications, 2000–2009 shows 7322 and 2010–2019 shows 23,426. With the continuous evolution of these technologies, it seemed appropriate that the OATech Network + should address the topic of the potential of technologies for subcategorising OA and it was felt that a Delphi meeting would be an appropriate approach.

## 2 Methods

This Delphi study consisted of a two-day focus group meeting (see programme in Supplementary Table 1), together with online surveys using ‘Google Forms’, to assess the level of agreement on a number of statements relating to OA and the use of different technologies (see Supplementary Table 2). The group consisted of a number of different specialists, all with expertise and significant interest in OA (Supplementary Table 3). A questionnaire was formulated based on the most widely used technologies and research tools which may aid subcategorisation of OA. The technologies were chosen by the organisers from their knowledge of the field and review of the literature, including a search performed for this study. Selected examples of OA categorisations were taken from the recent literature through primary searches (using Medline, EMBASE and PubMed with ‘definition of osteoarthritis’ as a search term) and articles known to the authors. Questions requiring free-text opinions of panel members were included in the questionnaire, for example, ‘were any questions missing’ and ‘what was their personal definition of OA?'. Answers to the latter were used to start discussions at the meeting and to assess the similarity of expert definition and understanding of OA. Expert consensus was reached for each statement when ≥80% participants agreed with the statement and rejected if ≤ 20% of participants agreed, as commonly used in previous Delphi studies [26].

The questionnaire was tested on 3 world leading experts in the field of OA (Professors Richard Loeser, Mary Goldring and Virginia Kraus) and modified slightly on their advice, before being sent to the Delphi panel electronically (Table 1). Panel members were asked if they agreed/disagreed with each of the statements. Round 1 was completed before the two day meeting. Talks were given at the start of the meeting by experts in the technologies presented in the Introduction. All statements in Round 1 were retained for Round 2, viewed ‘live’ on the Delphi Google Form; any questions/statements which did not reach consensus in Round 2 were discussed in fine detail with participants suggesting potential improvements to statements. Once unanimous agreement on the wording was achieved, the wording was altered in the survey for voting on in Round 3 at the end of day 2. These changes to wording are shown in Table 1.

**Table 1:** Statements used in the DELPHI and the percentage of participants who agreed with the statements at each Round.

DELPHI statement/Question		Round 1	Round 2	Modified question for round 3	Round 3	
		Percentage agreement with statement				
1	OA is a disease of i Bone ii Cartilage iii **Bone and cartilage**	i. 2.9 ii. 5.7 iii. **91.4**	i. 3.1 ii. 0 iii. **96.9**			
2	OA always involves other tissues in the joint in addition to bone and or cartilage	63.9	**87.9**	**OA involves other tissues in the joint in addition to bone and cartilage**	**100**	
3	Early OA needs categorising differently to ‘established OA	**86.1**	**87.9**	Panel decided not to take this question forward		
4	Osteoarthritis needs re-defining	65.7	69.7			
**5**	**OA is a continuum**	**88.6**	**97**			
**6**	**Subcategorising OA is useful**	**94.3**	**100**			
7	The definition of OA needs to be joint specific	55.6	69.7	The definition of OA needs to encompass joint specific differences	66.7	
8	OA phenotypes should rely on underlying mechanisms	73.5	**84.8**			
9	X-rays alone can be used to categorise OA phenotype	5.6	6.1			
10	The Kellgren-Lawrence (KL) is the most appropriate for categorising OA on X-ray	50	74.2	**There is a need for an improved scoring system than the Kellgren-Lawrence for X-rays**	**93.9**	
11	MRI has no role to play in categorising OA	2.8	9.1			
12	A universal OA categorisation system can be used for all clinical cases of OA	44.4	56.3	Panel decided not to take this question forward		
13	The same categorisation system for OA can be used in the clinic and or research studies	57.1	59.4	The same categorisation system for OA should be used in the clinic and or research studies	78.8	
**14**	**The latest technological advances can be used to improve OA subcategorisation**	**100**	**100**			
15	Please say if you agree or disagree that the application of the following technologies can improve clinical OA subcategorisation				Clinical	Research
	***Epigenomics***	**84.8**	**87.5**			
	***Genetic analysis***	**91.4**	**97**			
	***MRI***	**100**	**97**			
	*X-ray*	**82.9**	48.5			
	*Ultrasound*	58.8	66.7			
	***Metabolomics***	78.8	**90.6**			
	***Proteomics***	**87.9**	**93.8**		**87.9**	75.8
	***Wet biomarker analysis***	**97.1**	**93.8**		75.8	69.7
	***Machine learning (AI)***	**88.9**	**100**			
	***Activity monitoring***	68.6	**90.9**			
	***Clinical engineering***	72.2	**87.5**			
16	Different OA subcategorisation systems have been suggested in the literature recently. Please say if you agree or disagree with the following statements taken from the literature.					
	A . Examples of OA can be: Hip/knee/hip and or knee [45]	58.3	72.7		51.5	
	B . Pain, symptoms, clinical examination and X-rays are the most useful factors in diagnosing early OA [46]	45.7	36.4		42.4	
	C . Pain, psychological distress, radiographic severity, BMI, muscle strength, inflammation and comorbidities are all associated with clinically distinct OA phenotypes [47]	60	69.7		51.5	
	D . Minimal joint disease, malaligned, biochemical, chronic pain, inflammatory metabolic syndrome and bone and cartilage metabolism are all main phenotypes of OA [48]	61.8	72.7		48.5	
	E . Knee OA phenotype is defined by patient reported frequent knee pain, cartilage damage and the presence of degenerative meniscal tissue [49]	58.8	48.5		39.4	
	F . OA can be classified by symptomatic radiographic OA (primary criteria) and pain alone (secondary criterion).	31.4	24.2		36.4	

Bold text indicates statements reaching consensus.

The aims of the Delphi study were to determine, using a panel of experts, 1. whether novel and existing technologies could aid in the subcategorisation of patients with osteoarthritis (OA) and 2. whether there is good knowledge and awareness of these technologies. This could then help define what technology gaps exist to allow recommendations on the focus of future collaborative and cross disciplinary research.

### 2.1 Participant identification and inclusion

Experts were selected from a wide range of disciplines relevant to the field of OA. All 130 members of the OATech Network+ were invited to take part. The Delphi questionnaire was emailed to 36 potential Delphi panel experts, who were all active in the OA field and expressed an interest in attending the meeting. The minimum requirement for all invited experts was to complete all three rounds of the Delphi and attend the meeting.

## 3 Results

Thirty three experts responded and completed the Round 1 questionnaires and attended the meeting, so becoming the Delphi panel (Supplementary Table 3). This consisted of basic science researchers, orthopaedic surgeons, physiotherapists, rheumatologists, engineers, radiologists, veterinary researcher and a clinical efficacy researcher from the UK (n = 31), America (n = 1) and the Netherlands (n = 1). However, several members were multi-faceted, e.g. being clinically active and performing basic research and running clinical trials. The questionnaire showed 37% of the panel members were actively treating patients whilst 63% were not, but might have patient contact. Twenty seven percent of panel members had been working in the field of OA for 0–5 years, with 24% being involved for >20 years (Supplementary Figure 1). Although the Delphi panel was made up of a diverse group of experts, none were experts in Delphi methodology. However, several panel members had significant, relevant experience of the process to mitigate this limitation.

The wording in the statements and the results of the Delphi questionnaire over 3 rounds are shown in Table 1 and summaries of the definitions of OA provided by participants from different disciplines in Table 2. Not all panellists answered the question on defining OA as all questions were optional for panel members, so results are shown from those available, with only small variations between and within professions.

**Table 2:** Definitions of OA from different professions on the Delphi panel.

Profession	OA definition
Physiotherapists	A syndrome affecting the joints of the body
	Joint pathology leading to pain and functional limitation that involves genetics and epigenetic factors
Rheumatologists	Structural alteration of cartilage and bone in a joint which results in pain and loss of function
	A disease of the whole joint with distinct clinical and structural phenotypes
	A disease of many tissues of the joint including cartilage and bone, associated with pain or stiffness
	Osteoarthritis is a whole-joint disease, affecting articular and periarticular tissues. It has components of degeneration, regeneration and low-grade inflammation that differ in extent and clinical consequences between joints, disease stages and patients
Orthopaedic Surgeons	Structural and biological derangement of joint (that isn't rheumatoid/ankylosing spondylosis/psoriatic
	A painful condition involving changes in multiple tissues of the joint
Engineers	A disease of the joint, characterised by pain, loss of function and degeneration/progressive damage of structures in/around the joint
	Musculoskeletal disease possibly triggered by altered joint biomechanics and biological signalling leading to joint tissue degeneration, inflammation and pain
Radiologist	Degenerative joint change currently based on exclusion of other causes
Vet	Degenerative whole joint disease with an inflammatory component
Scientists (researcher)	Joint disease that results in cartilage degeneration, bone changes and pain
	Degenerative disorder of the joint
	A degenerative disease of the bone and cartilage. Can lead to cartilage loss, joint inflammation, changes in the bone and pain

∗The number of comments shown indicates the number of people who provided definitions in each profession.

None of the six categorisations of OA taken from recent literature reached consensus in any round (Table 1). Furthermore, 4 of the 6 literature-derived definitions demonstrated a decrease in agreement between Rounds 2 and 3 (following the face-to-face meeting).

**Fig. 1:**
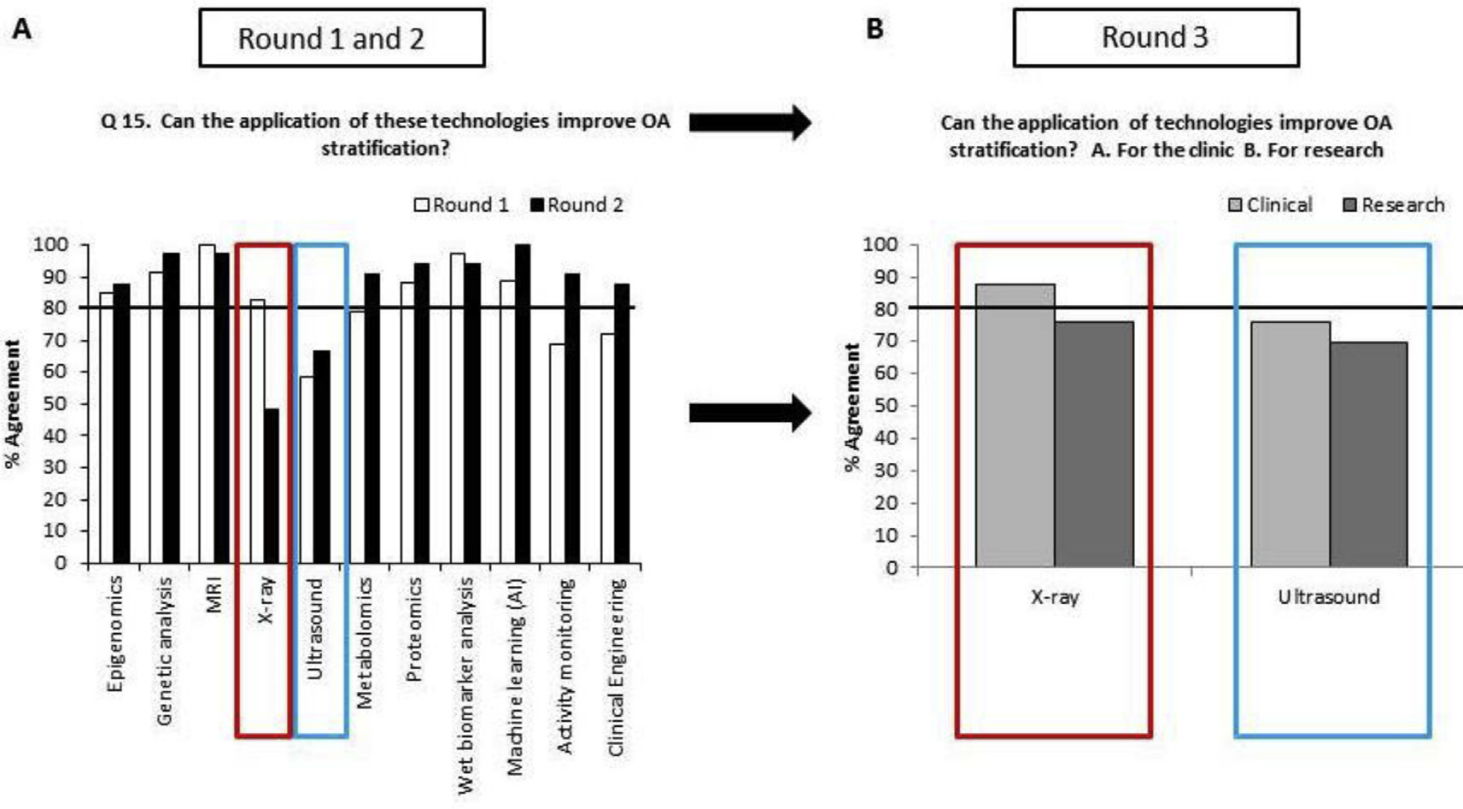
A. Frequency histogram indicating change of panel members' response as to whether different technologies were able to improve OA stratification in Round 1 (before the focus meeting) and Round 2 (after the instructive lectures at the start of the meeting). Nine of the 11 technologies reached consensus after the 2nd round. B. The modified question related to X-ray and ultrasound technologies for the 3rd round for the clinic and research and the percentage agreement.

In contrast, there was unanimous agreement in Rounds 1 & 2 that the latest technological advances could be used to improve OA subcategorisation (Table 1 & Fig. 1). Of the technologies identified, only the statement ‘X-rays alone can be used to categorise OA phenotype’ failed to reach consensus in Rounds 1 and 2, whilst there was no consensus in Round 2 for either X-rays or ultrasound as technologies which would to improve clinical OA subcategorisation (Table 1).

The technologies which gained greatest consensus in Round 2 for being of use in improving subcategorisation of OA were: ML (100%), genetic analysis and MRI (both 97%), proteomics and wet biomarker analysis (both 93.8%), activity monitoring (90.9%), metabolomics (both 90.6%), epigenomics and clinical engineering (both 88%). Eighty three percent of participants thought X-rays could aid subcategorisation of OA in Round 1, but this reduced to 49% in Round 2, whilst for ultrasound this changed from 59% in Round 1 to 67% in Round 2. Ultrasound was described as useful for identifying inflammation in the knee and could therefore be valuable in subcategorising OA, although some members did not feel that there was sufficient evidence presented to make an informed decision as this technology was not presented at the meeting.

There was much discussion on the usefulness of X-rays and the commonly used Kellgren-Lawrence (KL) score for staging disease. Discussions highlighted that radiography is considered outdated and flawed, but that X-rays are still the gold standard (alongside clinical criteria) for diagnosis and assessing OA in the clinic, e.g. for suitability for arthroplasty.

## 4 Discussion

Whilst OA has long been recognised as a heterogeneous multi-faceted disorder, progress into defining subgroups or categories has been poor; this is a likely reason why several clinical trials of novel pharmaceuticals or Disease Modifying Osteoarthritis Drugs (DMOADs) have failed [[27], [28], [29]]. In other areas of medicine such as asthma, subcategorisation has been achieved according to the pathological mechanisms (i.e. molecular endotyping) and clinical phenotyping [30]. It is to be hoped that this can be achieved for OA, resulting in improved diagnosis, understanding of disease mechanisms, identification of novel therapeutic targets, the development of new therapies and, subsequently better stratification and improved treatment of patients. Indeed, this was a conclusion of the inaugural meeting of an EPSRC-funded UK initiative for the OATech Network +, with the subsequent decision to utilise a Delphi-style process to address this topic.

As technology becomes more sophisticated and specialised there is a danger of working increasingly in silos. This process, including expert participants (>20% having >20 years’ OA research experience), from several disciplines, facilitated an appraisal across key areas where technology has made great advances. The benefits associated with this were indicated in participant feedback, for example, the change in consensus on technologies such as clinical engineering. The process highlighted a consensus belief that adopting key existing and emerging technologies (ML, genetic analysis, MRI, proteomics, wet biomarker analysis, activity monitoring, metabolomics, epigenomics and clinical engineering), would increase successful delivery of improved OA subcategorisation and discussions raised many suggestions as detailed below. In contrast, existing literature provided little agreement on the approach to OA categorisations and indeed, other studies that have highlighted the urgent need for updated definitions and categories [31,32].

X-rays, discussed at length, are well known to have limitations, especially with regard to the KL scoring system for radiographic diagnosis of OA [33,34]. The inclusion of clinical and non-clinical participants was particularly beneficial with orthopaedic surgeons highlighting that X-rays remain a valued clinical technology, being relatively simple, cheap, readily available and useful for diagnosis and treatment decisions. The KL radiographic classification scheme for OA, first described in 1957 [33], remains the most widely used clinical tool for the radiographic diagnosis of OA [34], despite its known limitations. Hence X-rays should be retained in OA studies, and based on previous improvements [35], the optimistic aim is to enhance their use through further application of ML and AI.

Epigenetic changes can modulate the impact of risk-conferring alleles of DNA polymorphisms that are associated with OA. For example, if a polymorphism is in a gene-regulatory element and the risk allele reduces gene expression, its effect can be attenuated or aggravated by DNA methylation of that element in an allele-specific manner [4]. As such, subgrouping OA patients by their genetic and epigenetic profile might reduce the heterogeneity seen across patients and enhance the interpretability of functional studies of genetic risk.

Large datasets generated from activity tracking through the increased adoption of smartphones and wearables, are likely to provide further opportunities to aid the stratification of OA populations. Activity monitoring research in OA populations has, in the past, been limited to measurements over short durations (i.e. up to 7-days), hence providing limited insight. Fitness trackers and smart phones have revolutionised the opportunities to collect continuous activity data more reliably and over longer time periods. Objective measures of physical activity can be used for monitoring recovery e.g. following joint arthroplasty, to measure short term recovery in terms of daily step count change over the first four weeks post-surgery [36]. Extending this approach over a large sample population would allow an expected trajectory of recovery to be developed such that patients deviating from it could, for example, be flagged for follow-up consultation. Deeper analysis and modelling of the inertial sensor data collected by wearables will be important for categorising OA populations. For example, multi-dimensional analyses of activity data have been found to be more accurately associated with functional test outcomes than step-count and sedentary time measures alone [37].

ML was the only technology reaching 100% consensus in its ability to improve OA subcategorisation in Round 2 of the Delphi, highlighting recognition of its potential usefulness. During discussions, the importance of integrating data, especially ‘big data', across disciplines and the application of ML approaches was highlighted as being of great importance. In big data science, ML is based on computer algorithms that can learn to identify complex patterns based on real data [38,39]. The goal of ML is to enable an algorithm to learn from past and/or present data and then to make predictions or decisions for unknown future events [40].

ML/AI is of paramount importance to all technologies generating ‘big data’, such as genomics, all omics and imaging modalities now used in biomarker and molecular signature discovery in OA. The use of ML/AI in integrating these advanced analytical techniques, provides the opportunity to build and test complex models incorporating important non-biomarker covariates. Multi-omics data has enabled biomarker generation for the stratification of patients into subgroups e.g. in oncology and other chronic diseases such as asthma [41,42]. This allows subcategorisation into groups based on genetic variability and other biomarkers so that medications may be tailored to individuals [43,44]. Big data systems using multi-omics (genomics, proteomics, metabolomics and epigenomics), enables understanding of interactions and functions of the genome, often identifying unexpected functions or possibly illustrating the interplay between the genome, the cellular environment and the progression of disease.

In summary, a Delphi-type exercise was undertaken as a route to obtaining expert consensus from a range of disciplines, regarding the role of novel experimental technology in OA research. It provided a valid route to recommendations for the focus and direction that should be adopted by the cross-disciplinary OA research community. Rather than employing individual technologies, it is likely that combining several identified technologies (eg proteomics, imaging and clinical engineering, together with machine learning), across sites, focussing on one or more OA subgroups will reap real benefits and provide important advances in the field of osteoarthritis research.

## Author contributions

All authors contributed to the ideas, questionnaire and writing the manuscript. All authors gave final approval of the version submitted.

## Role of the funding source

This meeting was funded by OATech Network+ (EP/N027264/1) and CM by Versus Arthritis Tissue Engineering and Regenerative Therapies Centre (Grant number 21156).

## Declaration of Competing Interest

The authors report that there is no conflict of interest.
