# Supplementary material for: The use of technology in the subcategorisation of osteoarthritis: a Delphi study approach
Source: Osteoarthr Cartil Open. 2020 Jun 9;2(3):100081. doi: 10.1016/j.ocarto.2020.100081 (PMC9718088; doi:10.1016/j.ocarto.2020.100081)
Supplement: Multimedia component 1 [file mmc1.docx]

**SUPPLEMENTARY DATA**

**Supplementary Figure 1** (this is being moved from the manuscript to Supplementary Figure on the suggestion of Reviewer 2).


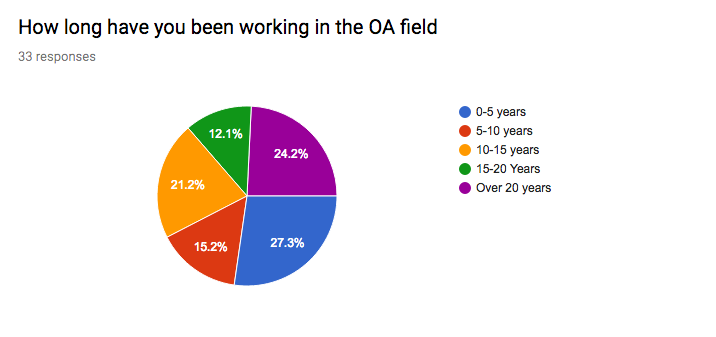


**Figure 1.** Pie chart indicating how long the DELPHI panel members have been working in the field of OA, taken from the results of the questionnaire.

**Supplementary** **Table 1**. The programme for the two-day Delphi meeting


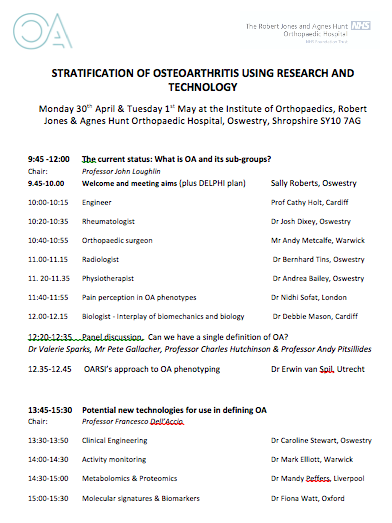


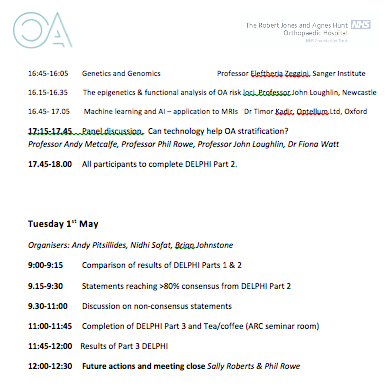


**Supplementary** **Table 2.** Basic definitions of the technologies discussed at the Delphi meeting

| \| **Technology** \| **Definition** \| \| --- \| --- \| \| Epigenomics \| Epigenetics is the study of changes in gene function that are heritable but do not involve changes in the genome; the genome-wide analysis of epigenetics is termed epigenomics. Unlike the genome of multi-cellular organisms which is identical and remains fairly static, the epigenome is very dynamic, varying from one cell type to another and responding to various signalling pathways^[1]^.  Genomic modifications that alter gene expression that cannot be attributed to modification of the primary DNA sequence and that are heritable mitotically and meiotically are classified as epigenetic modifications. DNA methylation and histone modification are among the best-characterised epigenetic processes^[2]^.  Epigenomics has only become possible in recent years because of the advent of various sequencing tools and technologies, such as DNA microarrays, whole-genome resequencing, and databases for studying entire genomes^[3]^. \| \| Genetic analysis \| Genetic analyses include molecular technologies such as PCR, RT-PCR, DNA-sequencing, DNA microarrays and cytogenetic methods such as karyotyping and fluorescence in situ hybridisation, which can identify changes in chromosomes, genes or proteins.  Automated DNA sequencing techniques coupled with advances in computerised data handling have transformed genetics, enabling rapid determination of the nucleotide sequences of entire genomes. The bioinformatics revolution has allowed evolutionary relationships between organisms to be traced at the genome level and gene function to be analysed at the cellular level. Genetic analysis provides the starting point for unravelling the complexities of every aspect of metabolism, function, and development^[4]^. \| \| Magnetic Resonance Imaging (MRI) \| MRI scanners use radio waves, strong magnetic fields and magnetic field gradients, to create very detailed images. MRI is better for examining organs and soft tissues than other scanning techniques such as computed tomography (CT) or x-ray.  In MRI certain atomic nuclei absorb radio frequency energy when placed in an external magnetic field; the resultant evolving spin polarisation can induce an RF signal in a radio frequency coil and be detected. Hydrogen atoms are most often used to generate a macroscopic polarisation that is detected by antennae close to the patient being imaged. Hydrogen atoms are abundant in most biological organisms, particularly in water and fat. For this reason, most MRI scans essentially map the location of water and fat in the body. Pulses of radio waves excite the nuclear spin energy transition, and magnetic field gradients localise the polarisation in space. By varying the parameters of the pulse sequence, different contrasts may be generated between tissues based on the relaxation properties of the hydrogen atoms^[5]^. \| \| X-ray imaging \| The radiological density, which affects how X-rays travel through tissues, is determined by both the density and the atomic number of the materials being imaged.  Hence bone and tissues containing calcium, readily absorb X-rays and produce a high contrast image on the x-ray detector, in comparison to other tissues. ^[6]^. Computer tomography (CT) utilises 3D X-ray imaging. \| \| Ultrasound \| Ultrasound imaging uses sound waves at frequencies above the audible range in human (>20,000 Hz- 4GiHz) The most common type of image produced is a Brightness-mode image, which displays the acoustic impedance of a two-dimensional cross-section of tissue, but the technology can also be used to illustrate tissue stiffness and motion , the presence of specific molecules, the location of blood and blood flow. Ultrasound has several advantages when compared to other medical imaging methods. It is highly portable, provides images in real time, it is lower in cost than other imaging modalities, non invasive and does not use harmful ionising radiation. Limitations include difficulty-imaging structures behind bone and air or gases^[7]^. \| \| Metabolomics \| Metabolomics is the systematic identification and quantification of the small molecule metabolic products (the metabolome) of a biological system (cell, tissue, organ, biological fluid, or organism) at a specific point in time. Mass spectrometry and NMR spectroscopy are the techniques most often used for metabolome profiling^[8]^. \| \| Proteomics \| Proteomics, usually assayed via high throughput technologies such as mass spectrometry, assesses the set of proteins produced in an organism or system, often in biological context.  Tandem-MS and gel-based techniques such as differential in-gel electrophoresis (DIGE) generate vast amounts of data, requiring databases and sophisticated computer programs to identify connections between their results and eg reference datasets ^[9]^. \| \| Wet biomarkers \| The National Institutes of Health (NIH) Biomarkers Definition Working Group defined a biomarker as “a characteristic that is objectively measured and evaluated as an indicator of normal biological processes, pathogenic processes, or pharmacologic responses to a therapeutic intervention”.   “Wet” biomarkers, usually measured in serum, plasma, synovial fluid or urine, may include proteins, protein fragments, metabolites or micro RNAs.. These are analysed via a number of techniques such as ELISA, proteomics or mass spectrometry ^[10, 11]^. \| \| Machine learning (ML) \| ML is a branch of artificial intelligence concerned with the construction of programs that learn from experience. Learning may take many forms, ranging from examples and analogy to autonomous learning of concepts and by discovery. Incremental learning involves continuous improvement as new data arrives while one-shot or batch learning distinguishes a training phase from the application phase. Supervised learning occurs when the training input has been explicitly labelled with the classes to be learned. Most methods aim to demonstrate generalisation whereby the system develops efficient and effective representations that encompass large amounts of closely related data^[12]^. \| \| Activity monitoring \| Activity monitoring usually utilises an instrument or device for checking, measuring, or keeping a record of a activity. Technology is rapidly advancing in terms of the measures and accuracy activity monitors can provide. On the lowest cost end of the scale, pedometers provide simple daily step count measures which are simple to use and fit. At the highest end of the scale research grade activity monitors all tend to use similar hardware, consisting of an accelerometer, gyroscope and magnetometer. These devices may differ in the features of the software and algorithms that can provide numerous activity related variables through to near complete gait analysis. (Mark Elliot, Personal communication). \| \| Clinical engineering \| This is a part of Biomedical Engineering focused on the applications of theories and methodologies of the broad biomedical engineering field to improve the quality of health services. It especially concerns the appropriate management of biomedical technologies and the development and the adjustment of hospital informative systems and telemedicine networks. Clinical Engineering combines with the medical knowledge for conducting of healthcare activities by providing expertise in a wide spectrum of topics, from human physiology and biomechanics to electronics and computer science^[13]^. \| |  |
| --- | --- | --- | --- | --- | --- | --- | --- | --- | --- | --- | --- | --- | --- | --- | --- | --- | --- | --- | --- | --- | --- | --- | --- | --- | --- |

**Supplementary data references**

1. Carlberg C, Molnár F. What Is Epigenomics?. Human Epigenomics. Springer, Singapore, 2008.
2. Russell P.J. iGenetics: A Molecular Approach (3rd ed.). San Francisco: Pearson
   Benjamin Cummings, 2010.
3. Bonetta L. Epigenomics: Detailed analysis. Nature 454 (2008) 795–798.
4. National Cancer Institute, genetic analysis.
   <https://www.cancer.gov/publications/dictionaries/cancer-terms/def/genetic-analysis> (accessed 22nd April 2020).
5. McRobbie D.W. MRI from picture to proton. Cambridge, UK; New York: Cambridge
   University Press. 2007, [ISBN](https://en.wikipedia.org/wiki/ISBN_(identifier)) [978-0-521-68384-5](https://en.wikipedia.org/wiki/Special:BookSources/978-0-521-68384-5).
6. National Institute for Biomedical imaging and Bioengineering, x-ray imaging. <https://www.nibib.nih.gov/science-education/science-topics/x-rays> (accessed 22nd
   April 2020).
7. Liu S, Wang Y, Lei B, Liu L, Xiang L, Ni D, Wang T. Deep Learning in Medical Ultrasound Analysis: A Review. Engineering. 5 (2019) 261-275.
8. Nature.com, metabolomics.
   <https://www.nature.com/subjects/metabolomics> (accessed 22nd April 2020).
9. EMBL-EBI, proteomics.
   <https://www.ebi.ac.uk/training/online/course/proteomics-introduction-ebi-resources/what->proteomics (accessed 28^th^ April 2020).
10. Bauer D.C, Hunter D.J, Abramson S.B, Attur M, Corr M, Felson D et al. Classification
    of osteoarthritis biomarkers: a proposed approach. Osteoarthritis Cartilage. 14 (2006) 723-727.
11. Henrotin Y, Sanchez C, Bay-Jensen A.C,^.^ Mobasheri A. Osteoarthritis biomarkers
    derived from cartilage extracellular matrix: Current status and future perspectives.
    Annals of Physical and Rehabilitation Medicine. 59 (2016) 145-148.
12. Oxford Reference, machine learning.
    <https://www.oxfordreference.com/view/10.1093/oi/authority.20110803100122832>
    (accessed 22^nd^ April 2020).
13. Derrico P, Ritrovato M, Nocchi F, Faggiano F, Capussotto C, Franchin T and De Vivo L. Clinical Engineering, in: G.D. Gargiulo, A. McEwan (Eds.), Applied Biomedical Engineering, IntechOpen, 2011, DOI: 10.5772/19763.

**Supplementary Table 3**. Individuals taking part in the Delphi exercise

|  | **Profession** | **Qualifications** | **Location** | **DELPHI Panel member Y/N*** |
| --- | --- | --- | --- | --- |
| **Clinicians** | | | | |
| 1 | Orthopaedic Surgeon | MBChB FRCS (T&O) | Oswestry | Y |
| 2 | Orthopaedic surgeon | MBChB (Hons), MRCS (Ed), FRCS (T&O) | Oswestry | Y |
| 3 | Orthopaedic surgeon | FRCS(T&O) Ph.D | Warwick | N |
| 4 | Physiotherapist | Ph.D | Oswestry | Y |
| 5 | Physiotherapist/scientist | Ph.D | Edinburgh | Y |
| 6 | Physiotherapist/scientist | Ph.D | Cardiff | Y |
| 7 | Radiologist | Dr Med, FRCR | Oswestry | Y |
| 8 | Radiologist /  Imaging scientist | MB, ChB, MD | Warwick | Y |
| 9 | Rheumatologist | MB BS, MRCP, MD, FRCP | Oswestry | N |
| 10 | Rheumatologist/Scientist | MD, Ph.D | QMUL, London | Y |
| 11 | Rheumatologist/Scientist | MD, Ph.D | St Georges London | Y |
| 12 | Rheumatologist/Scientist | MD, Ph.D | Utrecht | N |
| 13 | Rheumatologist/Scientist | MBBS, Ph.D, FRCP | Oxford | Y |
| 14 | Scientist/Veterinary Practitioner | BVetMed MRCVS, Ph.D | Liverpool | Y |
| **Scientists** | | | | |
| 1 | Bioengineer/statistician | Ph.D | Oswestry | Y |
| 2 | Bioengineer | Ph.D | Oxford | Y |
| 3 | Engineer | Ph.D | Cardiff | Y |
| 4 | Engineer | Ph.D | Cardiff | Y |
| 5 | Biomedical Engineer | Ph.D, PGDip, CEng, FIMechE | UCL, London | Y |
| 6 | Biomedical Engineer | MEng, Ph.D | Strathclyde | Y |
| 7 | Clinical Engineer | Ph.D | Oswestry | Y |
| 8 | Biotech Engineer (PDRA) | Ph.D | Edinburgh | Y |
| 9 | Scientist, Bone biologist | Ph.D | Sheffield | Y |
| 10 | Scientist, Cell biologist | Ph.D | Oswestry | Y |
| 11 | Scientist, Cell biologist | Ph.D | Oswestry | Y |
| 12 | Scientist, Cell biologist | Ph.D | Oswestry | Y |
| 13 | Scientist | Ph.D | RJAH | Y |
| 14 | Scientist, Cell biologist | Ph.D | QMUL, London | Y |
| 15 | Scientist, Cell biologist | Ph.D | OHSU Oregon | Y |
| 16 | Scientist, Digital healthcare | Ph.D | Warwick | Y |
| 17 | Imaging scientist | Ph.D | Oxford | Y |
| 18 | Scientist, Mathematician | Ph.D | Oxford | N |
| 19 | Scientist, Geneticist | PhD | Newcastle | Y |
| 20 | Scientist | Ph.D | Helmholtz Zentrum Munich | N |
| 21 | Scientist, MSK biologist | Ph.D | Cardiff | N |
| 22 | Scientist, MSK biologist | Ph.D | RVC London | Y |
| 23 | PhD Student | BSc (Hons) | Oswestry | Y |
| 24 | PhD Student/trainee orthopaedic surgeon | MD | Oswestry | Y |

* Listed participants who were not part of the Delphi panel are either some of the speakers or attendees of only part of the meeting.
